# Accuracy of the Apple Watch Series 9 for Measures of Energy Expenditure and Heart Rate at Rest and During Exercise: Impact of Skin Pigmentation

**DOI:** 10.3390/jfmk9040275

**Published:** 2024-12-17

**Authors:** Sydney E. Chase, Rebecca G. Liddell, Chloe L. McGonagle, Stephen J. Ives

**Affiliations:** Health & Human Physiological Sciences, Skidmore College, Saratoga Springs, NY 12866, USA; schase@skidmore.edu (S.E.C.); rliddell@skidmore.edu (R.G.L.); cmcgonag@skidmore.edu (C.L.M.)

**Keywords:** energy expenditure, heart rate, fitness tracking, activity, monitoring

## Abstract

Background: The Apple Watch provides promising health data that could aid in increasing exercise adherence; regular exercise can help individuals manage and prevent diseases such as obesity and cardiovascular disease. However, the impact of skin pigmentation on the accuracy of the Apple Watch Series 9 for measures of energy expenditure (EE) and heart rate (HR) is unknown. Purpose: The purpose of this study was to determine the accuracy of the Apple Watch Series 9 on various skin pigmentations for measures of EE and HR. Methods: Thirty young, healthy individuals were assigned to one of three groups based on their scores on the Fitzpatrick skin survey. Participants completed a 10 min treadmill protocol with varying speeds and inclines while wearing an Apple Watch Series 9, a two-way non-rebreathing mouthpiece connected to a Parvo Medics metabolic cart, and a Polar H7 chest strap to measure EE and HR. Results: Overall, EE was found to be inconsistent for all skin pigmentation groups. However, for HR, the Apple Watch Series 9 was more variable (i.e., less accurate) for darker skin pigmentations compared to lighter skin pigmentations. Conclusions: The Apple Watch Series 9 was found to vary in both EE and HR measures from criterion across intensity and skin pigmentation, with greater discrepancies for individuals in Group 3 for measures of HR. Further investigation might aim to study the impact of skin pigmentations and wrist subcutaneous fat on the accuracy of the latest Apple Watch Series 9 for measures of EE and HR.

## 1. Introduction

“Exercise is medicine” is an expression that physicians, health professionals, and educators promote [1]. The World Health Organization (WHO) defines physical activity as “any bodily movement produced by skeletal muscles that requires energy expenditure” [2]. Continuing regular physical activity can help individuals prevent and manage diseases such as obesity, stroke, cancer, heart disease, and hypertension and improve their mental health and overall quality of life [3]. However, as often as exercise is promoted for its beneficial effects on health, there has been an increase in lack of adherence from individuals, and thus, increased risks of adverse health outcomes such as obesity and cardiovascular disease [4].

As death rates from obesity and CVD increase around the world, it is imperative for individuals to have an easily accessible way to track their health. To this end, multifunctional fitness devices such as the Apple Watch, Fitbit, and Garmin have been developed. Although there have been many different brands of fitness devices on the market, Apple Watches have increased in popularity. One in five people in the United States regularly wears the device, which was the best-selling wearable fitness device in 2020, with 33.9 million watches sold [5].

Although these devices are increasing in popularity and have many valuable functions, there are concerns regarding the accuracy of the sensors for differing skin pigmentations. Health disparities are defined by differences in the burden of disease, violence, injury, or opportunities for adequate health care for a socially disadvantaged population [6]. Unequal medical care for patients of color is caused by decades of the majority of clinical studies only investigating white males and a limited understanding of social determinants of illness [7]. However, there have been recent efforts to increase the representation of Black, Hispanic, and female individuals in clinical studies. Nevertheless, data have shown that racial and ethnic minority groups experience higher rates of death from diabetes, obesity, asthma, hypertension, and heart disease compared to white individuals [8].

Previous studies have found that the pulse oximeter, which is a widely used medical device, provides less accurate results when assessing those of darker skin pigmentations [9]. This was found to be due to the interference of the melanin with the quality of the reflected signal from the sensors. The inaccuracy of the pulse oximeter for individuals with darker skin pigmentations goes back to 1976, when reading errors showed lower blood oxygen saturation values for darker-skinned patients. However, more recent studies have shown that pulse oximeters overestimate blood oxygen saturation levels for darker skin pigmentations [9]. This raises concern in the medical world, as it can increase the incidence of occult hypoxemia. This can be harmful, as occult hypoxemia is defined as low blood oxygen levels that are not detected by a pulse oximeter. This can lead to worsening health conditions and delayed or inadequate treatment [9]. Previous research on the impact of skin pigmentation on the accuracy of the pulse oximeter was likely the impetus for Apple to remove this feature from the Apple Watch; however, the heart rate sensor, like many others, uses photoplethysmography (PPG) or optical sensors for the detection of heart rate and thus may be susceptible to the same bias.

The current reality regarding health disparities for those of differing skin pigmentation raises the following question: does skin pigmentation impact the accuracy of Apple Watch Series 9 EE and HR measurements? The purpose of this study was to determine the accuracy of the Apple Watch Series 9 on various skin pigmentations for measures of EE and HR in a heterogenous sample. Second, we aimed to delineate the potential impact of skin pigmentation on the differences and relations between the Apple Watch Series 9 and standard equipment. It was hypothesized that the Apple Watch Series 9 is not an accurate measuring device for EE and HR. Further, it was hypothesized that the Apple Watch Series 9 is less accurate for darker skin pigmentations (Group 3) compared to lighter skin pigmentations (Group 1 and 2) in measures of EE and HR.

## 2. Materials and Methods

**Figure 1:**
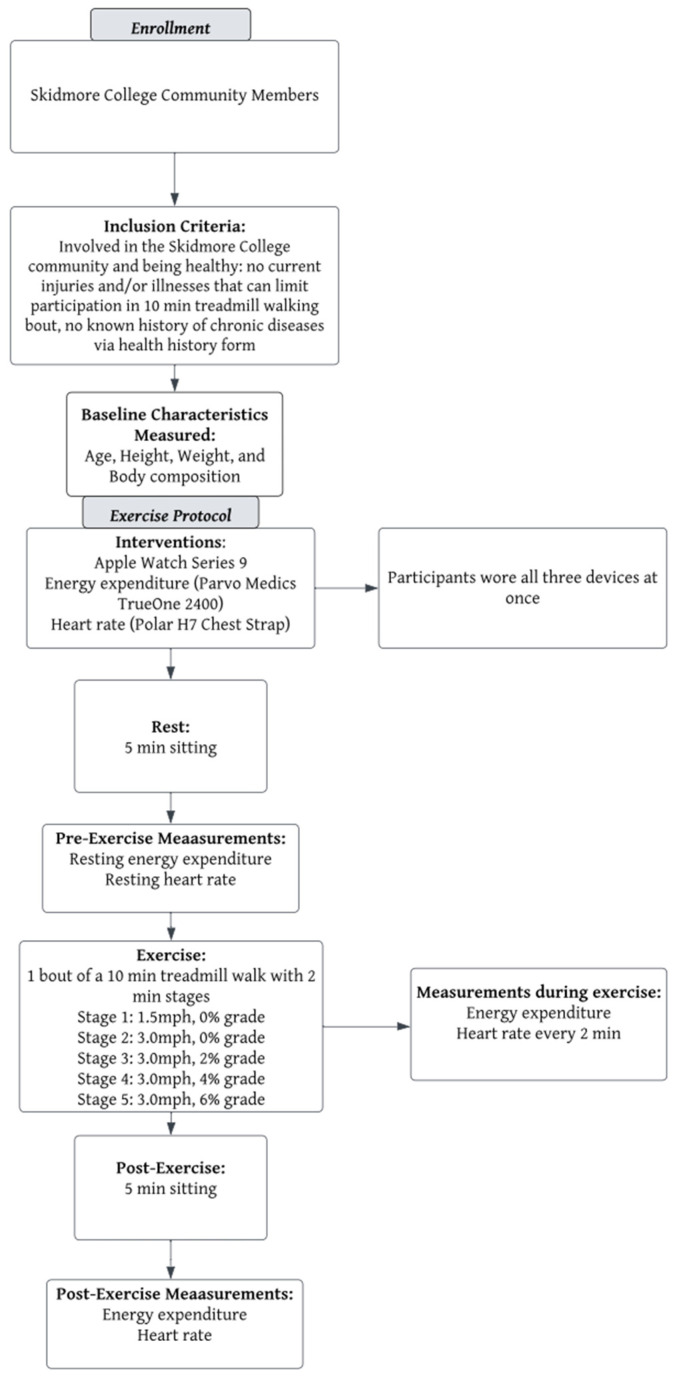
Overview of the experimental design.

Participants at least 18 years old were recruited from the Skidmore College community via email. The schematic of the experimental design is illustrated in Figure 1. Participants volunteered to join the study if they met the following inclusion criteria: being healthy—which included no current injuries and/or illnesses that might impact an acute bout of treadmill walking—and no known history of chronic disease, assessed via health history form. Participants were notified of the possible risks and benefits of this study and signed the informed consent form. This study was reviewed and approved by the Skidmore College Institutional Review Board for the use of human participants in research (IRB#2311-1121) and was conducted in accordance with the most recent revisions to the Declaration of Helsinki. There was a total of 34 participants recruited from the Skidmore College Community for this study. However, due to three no responses and one participant not meeting the inclusion criteria, a total of 30 college-aged participants completed this study. Initial sample size estimation in a correlation model, with alpha = 0.05 and an effect size of 0.5 (large), to achieve a power of 0.8, 23 individuals would be needed (G*Power, Dusseldorf, Germany).

### 2.1. Procedures

Upon arrival to the laboratory, participants’ baseline characteristics such as age in years, height using a stadiometer in centimeters (Seca, Mt. Pleasant, SC, USA), weight using a scale in kilograms, and body composition using bioelectrical impedance analysis (BIA) in body fat percent (RD-545, Tanita, Arlington Heights, IL, USA) [10,11,12] were measured. Each participant was asked to fill out a Fitzpatrick phenotype questionnaire via a Google form before they began testing to be placed into one of the three skin pigmentation groups: Group 1, score of 0–13 (skin pigmentation I, II); Group 2, score of 14–27 (skin pigmentation III, IV); and Group 3, score of 28–35+ (skin pigmentation V, VI). The Fitzpatrick scale has been documented as a reliable method of skin type determination in adolescents [13].

Participants were instrumented with an Apple Watch Series 9 (Apple Inc., Cupertino, CA, USA) that was updated with their age, body weight, and height prior to testing. Participants were asked to place the Apple Watch Series 9 on their left wrist to compare to the measure criterions for EE using a two-way non-rebreathing mouthpiece connected to a Parvo Medics True One 2400 metabolic cart (Parvo Medics, Salt Lake City, UT, USA) [14] and for HR wearing a Polar H7 chest-strap HR monitor (Polar Electro, Kempele, Finland) [15]. Before participating in the 10 min exercise protocol, participants were seated for 5 min to collect resting EE and HR. Participants were asked to walk on a treadmill (TMX-425, Trackmaster, Newton, KS, USA) for a total of 10 min, with 2 min stages of the corresponding speeds and incline grades: 1.5 mph 0% grade, 3 mph 0% grade, 3 mph 2% grade, 3 mph 4% grade, and 3 mph 6% grade. HR was collected continuously but recorded once at each timepoint at the end of each 2 min stage, while EE was collected for the entire final min (again 15 s averages) of each stage and subtracted from the total EE of the previous stage’s final min. Following the exercise protocol, participants were seated for 5 min, where EE and HR were measured.

### 2.2. Statistical Analysis

All statistical analyses were performed using open-sourced JASP software (v.0 17.3, Amsterdam, The Netherlands). A paired-samples *t*-test was used to determine differences between the Apple Watch Series 9 and criterion measures. A Shapiro–Wilks test was used to assess the normality of the data; if a violation was found, a non-parametric Wilcoxon signed rank test was conducted. A one-way analysis of variance (ANOVA) test was used to determine differences in participant characteristics across three groups: Group 1, Group 2, and Group 3. A Levene’s test was used to assess the normality of the data. If a violation was found, a non-parametric Welch test was applied to the degrees of freedom. If a significant interaction was found, a Tukey’s honestly significant difference (HSD) post-hoc test was used to make pairwise comparisons. A two-way mixed analysis of variance (RMANOVA) test was used to determine the potential main effect of skin pigmentation classification, the potential main effect of time, and their potential interaction (group and time). A sphericity test was conducted to assess the normality of the data. If a violation was found, a correction of a Greenhouse–Geisser test was used to adjust the degrees of freedom. If a significant interaction was found, then a Holm post-hoc test was conducted to determine where the difference was located between the variables. For the ANOVA tests, a partial eta squared (ηp2) was used to estimate effect size, where 0.01, 0.06, and 0.14 indicated small, medium, and large effect size, respectively [16]. For conducted post-hoc tests, a Cohen’s d was used to estimate effect size where 0.2, 0.5, and 0.8 were expressed as small, moderate, or large effects, respectively. A Pearson’s correlation analysis was used to determine the relationship between variables. A pairwise Shapiro test was conducted to assess the normality of the data’s distributions. If a violation was found, then Spearman’s rho was used as a non-parametric test. An r value was used as an estimate of effect size. The strength of the relationship is defined as: r values of 0.00–0.10, 0.10–0.39, 0.40–0.69, 0.70–0.89, and 0.90–1.00 interpreted as negligible, weak, moderate, strong, or very strong correlation, respectively [17]. To further analyze the comparison between measurement methods, simple bivariate regression analysis was performed, as well as Bland–Altman plots. The level of significance was set as *p* < 0.05. All data presented as means ± standard deviation, unless noted otherwise.

## 3. Results

Table 1 displays the descriptive characteristics of the 30 college-aged participants from Skidmore College. Table 2 displays the pairwise comparison between the Parvo Medics and Apple Watch Series 9 on measures of EE. Table 3 displays the pairwise comparison between the Polar H7 and Apple Watch Series 9 on measures of HR. Table 4 displays a pairwise comparison between the Parvo Medics and Apple Watch Series 9 on measures of EE for each group. Table 5 displays a pairwise comparison between the Polar H7 and Apple Watch Series 9 on measures of HR for each group.

### 3.1. t-Test Comparisons of Devices

For all skin pigmentation groups (N = 30), the Apple Watch Series 9 significantly underestimated compared to the Parvo Medics for resting EE (*p* < 0.001, Cohen’s D = −1.000; Table 2), min 0 (*p* < 0.001, Cohen’s D = −1.214), and post-exercise EE (*p* < 0.001, Cohen’s D = −5.253). Further, the Apple Watch Series 9 significantly overestimated compared to the Parvo Medics for EE at min 2 (*p* < 0.001, Cohen’s D = −1.000). There was no significant difference between the Parvo Medics and Apple Watch Series 9 for EE measures at min 4 (*p* = 0.136, Cohen’s D = 0.280), min 6 (*p* = 0.554, Cohen’s D = 0.109), min 8 (*p* = 0.293, Cohen’s D = 0.195), and min 10 (*p* = 0.258, Cohen’s D = 0.211).

For all skin pigmentation groups, the Apple Watch Series 9 significantly underestimated HR for min 4 compared to the Polar H7 (*p* = 0.007, Cohen’s D = −0.570; Table 3) and min 6 (*p* = 0.020, Cohen’s D = −0.554). There was no significant difference between the Polar H7 and Apple Watch Series 9 measures for HR at rest (*p* = 0.736, Cohen’s D = 0.077), min 0 (*p* = 0.523, Cohen’s D = −0.135), min 2 (*p* = 0.212, Cohen’s D = 0.275), min 8 (*p* = 0.054, Cohen’s D = −0.433), min 10 (*p* = 0.271, Cohen’s D = −0.264), and post rest (*p* = 0.819, Cohen’s D = −0.053).

There was no significant difference between the Apple Watch Series 9 and Parvo Medics EE for Group 1 (*p* = 0.168, Cohen’s D = −0.212; Table 4). The Apple Watch Series 9 significantly underestimated compared to Parvo Medics EE for Group 2 (*p* = 0.011, Cohen’s D = −0.252) and Group 3 (*p* < 0.001, Cohen’s D = −0.735).

There was no significant difference between the Apple Watch Series 9 and Polar H7 for Group 1 (*p* = 0.088, Cohen’s D = −0.232; Table 5) and Group 2 (*p* = 0.739, Cohen’s D = 0.035). The Apple Watch Series 9 significantly underestimated compared to the Polar H7 HR for Group 3 (*p* = 0.014, Cohen’s D = −0.431).

### 3.2. ANOVA Assessment of Groups over Time by Device

**Figure 2:**
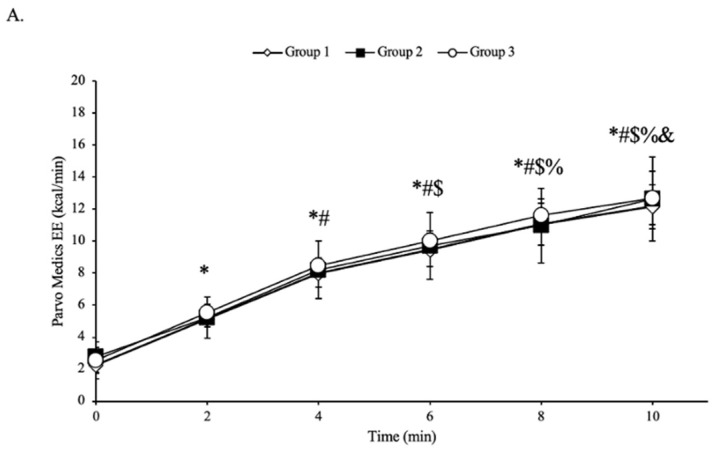

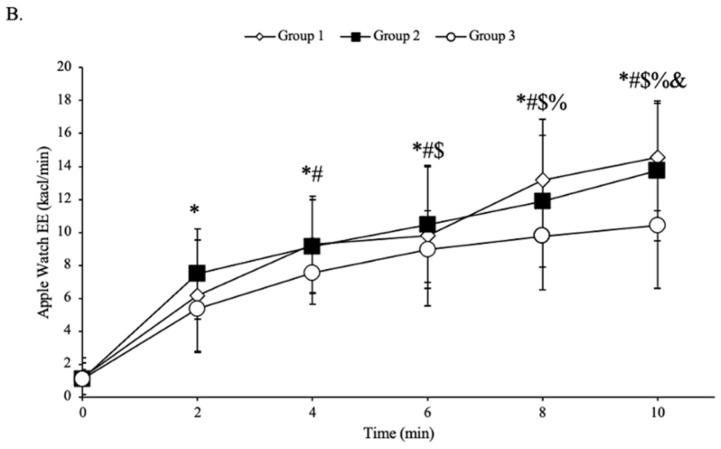
Energy expenditure response in calories per min (kcal/min) of the college-aged participants (N = 30) from the Parvo Medics (panel **A**) or the Apple Watch Series 9 (panel **B**) during each stage of the 10-min treadmill protocol. Data presented as means ± standard deviation. * Significant difference from 0 min (*p* < 0.05). # Significant difference from 2 min (*p* < 0.05). $ Significant difference from 4 min (*p* < 0.05). % Significant difference from 6 min (*p* < 0.05). & Significant difference from 8 min (*p* < 0.05).

For all groups (N = 30), there was no interaction of skin pigmentation groups (Group I, II, and III) over time (min 0, 2,4,6,8, and 10) in measures of EE from the metabolic cart (*p* = 0.849, η^2^p = 0.025; Figure 2A). There was no main effect of skin pigmentation groups on EE from the metabolic cart (*p* = 0.836, η^2^p = 0.013). There was a main effect of time on the EE (*p* < 0.001, η^2^p = 0.948), with the expected increases in response to exercise. For all groups, there was no significant interaction between time and skin pigmentation groups for measures of EE taken from the Apple Watch Series 9 (*p* = 0.205, η^2^p = 0.100; Figure 2B). There was no main effect of skin pigmentation groups (*p* = 0.332, η^2^p = 0.079). There was a main effect of time for EE (*p* < 0.001, η^2^p = 0.813), with the expected increases in response to exercise.

**Figure 3:**
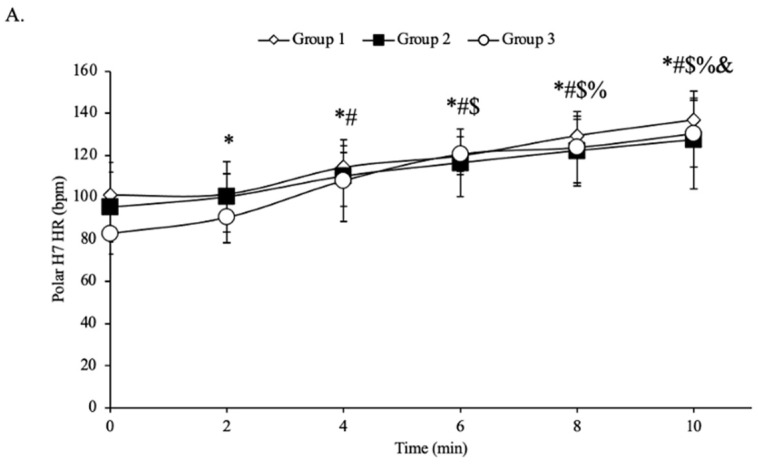

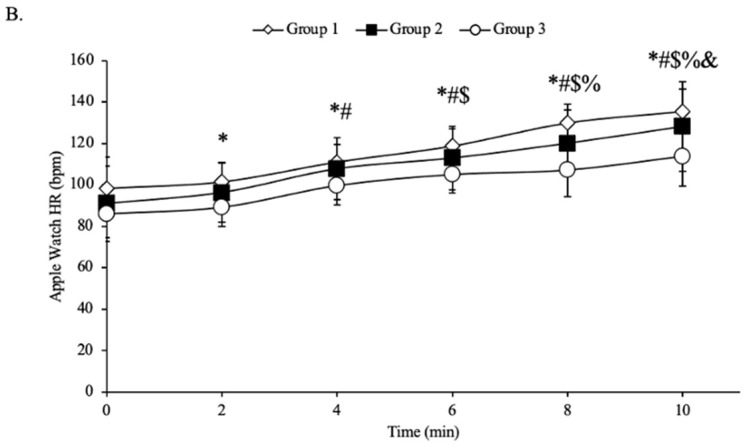
Heart rate response in beats per min (bpm) of the college-aged participants (N = 30) from the Polar H7 (panel **A**) or the Apple Watch Series 9 (panel **B**) during each stage of the 10-min treadmill protocol. Data presented as means ± standard deviation. * Significant difference from 0 min (*p* < 0.05). # Significant difference from 2 min (*p* < 0.05). $ Significant difference from 4 min (*p* < 0.05). % Significant difference from 6 min (*p* < 0.05). & Significant difference from 8 min (*p* < 0.05).

For all groups, there was no main interaction between time and skin pigmentation groups in HR responses from Polar H7 (*p* = 0.332, η^2^p = 0.080; Figure 3A). There was no main effect of skin pigmentation groups on HR (*p* = 0.494, η^2^p = 0.051). There was a main effect of time on HR (*p* < 0.001, η^2^p = 0.658), with the expected increases in response to exercise. For all groups, there was no interaction between time and skin pigmentation groups on HR response measured from the Apple Watch Series 9 (*p* = 0.244, η^2^p = 0.094; Figure 3B). There was no main effect of skin pigmentation group on HR (*p* = 0.139, η^2^p = 0.136). There was a main effect of time on HR (*p* < 0.001, η^2^p = 0.815), with the expected increases in response to exercise.

### 3.3. Bland–Altman—EE and HR

**Figure 4:**
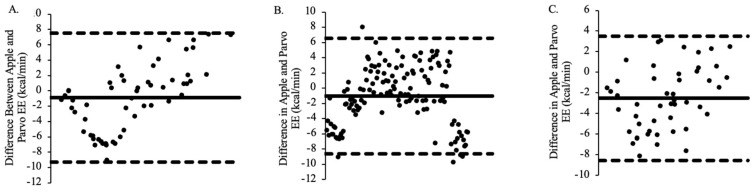
The agreement between Apple Watch Series 9 and Parvo Medics EE for all timepoints for Group 1 (panel **A**; M = −0.884), Group 2 (panel **B**; M = −1.020), and Group 3 (panel **C**; M = −8.529).

When comparing Apple Watch Series 9-measured EE to the Parvo Medics-measured EE for Group 1, a mean bias of −0.88 was obtained, with 95% limits of agreement ranging from −9.28 kcal/min to 7.52 kcal/min (Figure 4A). When comparing Apple Watch Series 9-measured EE to the Parvo Medics-measured EE for Group 2, a mean bias of −1.02 was obtained, with 95% limits of agreement ranging from −8.62 kcal/min to 6.58 kcal/min (Figure 4B). When comparing Apple Watch Series 9-measured EE to the Parvo Medics-measured EE for Group 3, a mean bias of −2.5 was obtained, with 95% limits of agreement ranging from −8.53 kcal/min to 3.52 kcal/min (Figure 4C).

**Figure 5:**
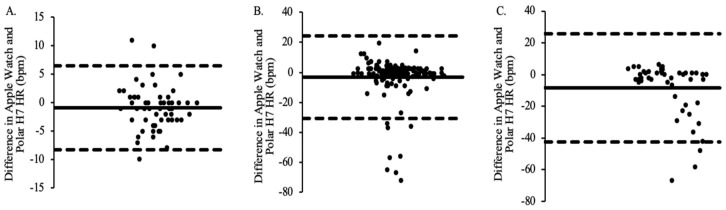
The agreement between Apple Watch Series 9 and Polar H7 HR for all time points for Group 1 (panel **A**; M = −0.875), Group 2 (panel **B**; M = −3.169), and Group 3 (panel **C**; M = −8.354).

When comparing Apple Watch Series 9-measured HR to the Polar H7-measured HR for Group 1, a mean bias of −0.88 was obtained, with 95% limits of agreement ranging from −8.28 bpm to 6.53 bpm (Figure 5A). When comparing Apple Watch Series 9-measured HR to the Polar H7-measured HR for Group 2, a mean bias of −3.17 was obtained, with 95% limits of agreement ranging from −30.73 bpm to 24.39 bpm (Figure 5B). When comparing Apple Watch Series 9-measured HR to the Polar H7-measured HR for Group 3, a mean bias of −8.35 was obtained, with 95% limits of agreement ranging from −42.26 bpm to 25.55 bpm (Figure 5C).

### 3.4. Correlations—EE and HR

**Figure 6:**
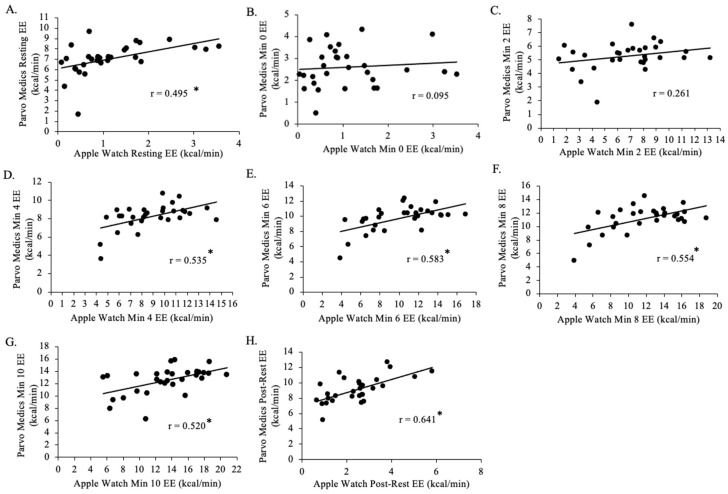
The relationship between the Apple Watch Series 9 and Parvo Medics on measures of energy expenditure (EE) for rest (panel **A**; r = 0.495; *p* < 0.001), 0 min (panel **B**; r = 0.095; *p* = 0.430), 2 min (panel **C**; r = 0.261; *p* = 0.220), 4 min (panel **D**; r = 0.535; *p* = 0.017), 6 min (panel **E**; r = 0.585; *p* = 0.002), 8 min (panel **F**; r = 0.554; *p* = 0.048), 10 min (panel **G**; r = 0.520; *p* = 0.003), and post-rest (panel **H**; r = 0.641; *p* < 0.001) in the participants in the study (N = 30). Data presented as values. * Statistical significance *p* < 0.05.

There was a positive negligible relationship between min 0 Parvo Medics-measured EE and min 0 Apple Watch Series 9-measured EE for all groups (N = 30, *p* = 0.430, r = 0.095; Figure 6). There was a positive weak relationship between min 2 Parvo Medics-measured EE and min 2 Apple Watch Series 9-measured EE for all groups (*p* = 0.22, r = 0.261). There was a positive moderate relationship between resting Parvo Medics-measured EE and resting Apple Watch Series 9-measured EE for all groups (*p* < 0.001, r = 0.495), between min 4 Parvo Medics-measured EE and min 4 Apple Watch Series 9-measured EE for all groups (*p* = 0.017, r = 0.535), between min 6 Parvo Medics-measured EE and min 6 Apple Watch Series 9-measured EE for all groups (*p* = 0.002, r = 0.583), between min 8 Parvo Medics-measured EE and min 8 Apple Watch Series 9-measured EE for all groups (*p* = 0.048, r = 0.554), between min 10 Parvo Medics-measured EE and min 10 Apple Watch Series 9-measured EE for all groups (*p* = 0.003, r = 0.520), and between post-rest Parvo Medics-measured EE and post-rest Apple Watch Series 9-measured EE for all groups (*p* < 0.001, r = 0.641).

**Figure 7:**
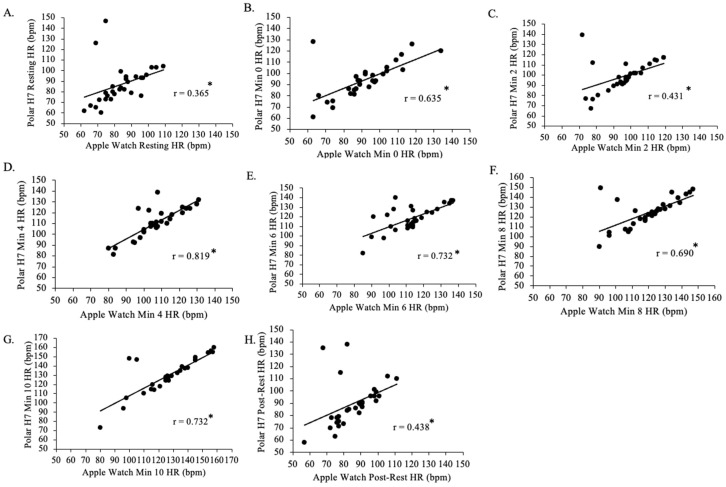
The relationship between the Apple Watch Series 9 and Polar H7 on measures of heart rate (HR) at rest (panel **A**; r = 0.365; *p* < 0.001), 0 min (panel **B**; r = 0.635; *p* < 0.001), 2 min (panel **C**; r = 0.431; *p* < 0.001), 4 min (panel **D**; r = 0.819; *p* < 0.001), 6 min (panel **E**; r = 0.732; *p* < 0.001), 8 min (panel **F**; r = 0.690; *p* < 0.001), 10 min (panel **G**; r = 0.732; *p* < 0.001), and post-rest (panel **H**; r = 0.438; *p* < 0.001) in the participants in the study (N = 30). Data presented as values. * Statistical significance *p* < 0.05.

There was a positive weak relationship between resting Polar H7-measured HR and resting Apple Watch Series 9-measured HR for all groups (*p* < 0.001, r = 0.365; Figure 7). There was a positive moderate relationship between min 0 Polar H7-measured HR and min 0 Apple Watch Series 9-measured HR for all groups (*p* < 0.001, r = 0.635), between min 2 Polar -measured HR and min 2 Apple Watch Series 9-measured HR for all groups (*p* < 0.001, r = 0.431), between min 8 Polar H7-measured HR and min 8 Apple Watch Series 9-measured HR for all groups (*p* < 0.001, r = 0.690), and between post-rest Polar H7-measured HR and post-rest Apple Watch Series 9-measured HR for all groups (*p* < 0.001, r = 0.438). There was a positive strong relationship between min 4 Polar H7-measured HR and min 4 Apple Watch Series 9-measured HR for all groups (*p* < 0.001, r = 0.819), between min 6 Polar H7-measured HR and min 6 Apple Watch Series 9-measured HR for all groups (*p* < 0.001, r = 0.732), and between min 10 Polar H7-measured HR and min 10 Apple Watch Series 9-measured HR for all groups (*p* < 0.001, r = 0.732).

#### 3.4.1. Group 1 Correlations—HR

**Figure 8:**
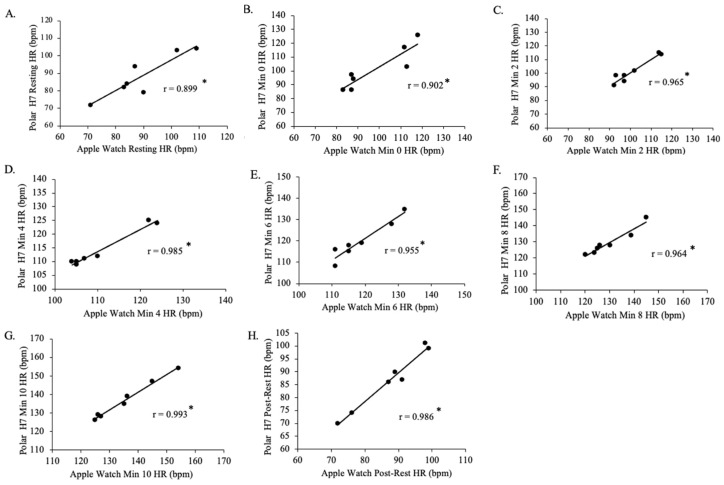
The relationship between the Apple Watch Series 9 and Polar H7 on measures of heart rate (HR) at rest (panel **A**; r = 0.899; *p* = 0.006), 0 min (panel **B**; r = 0.902; *p* = 0.005), 2 min (panel **C**; r = 0.965; *p* < 0.001), 4 min (panel **D**; r = 0.985; *p* < 0.001), 6 min (panel **E**; r = 0.955; *p* < 0.001), 8 min (panel **F**; r = 0.964; *p* < 0.001), 10 min (panel **G**; r = 0.993; *p* < 0.001), and post-rest (panel **H**; r = 0.986; *p* < 0.001) in the Group 1 participants in the study (n = 7). Data presented as values. * Statistical significance *p* < 0.05.

There was a positive strong relationship between resting Polar H7-measured HR and resting Apple Watch Series 9-measured HR for Group 1 (n = 7, *p* = 0.006, r = 0.899; Figure 8). There was a positive, very strong relationship between min 0 Polar H7-measured HR and min 0 Apple Watch Series 9-measured HR for Group 1 (n = 7, *p* = 0.005, r = 0.902), between min 2 Polar H7-measured HR and min 2 Apple Watch Series 9-measured HR for Group 1 (*p* < 0.001, r = 0.965), between min 4 Polar H7-measured HR and min 4 Apple Watch Series 9-measured HR for Group 1 (*p* < 0.001, r = 0.985), between min 6 Polar H7-measured HR and min 6 Apple Watch Series 9-measured HR for Group 1 (*p* < 0.001, r = 0.955), between min 8 Polar H7-measured HR and min 8 Apple Watch Series 9-measured HR for Group 1 (*p* < 0.001, r = 0.964), between min 10 Polar H7-measured HR and min 10 Apple Watch Series 9-measured HR for Group 1 (*p* < 0.001, r = 0.993), and between post rest Polar H7-measured HR and post rest Apple Watch Series 9-measured HR for Group 1 (*p* < 0.001, r = 0.986).

#### 3.4.2. Group 2 Correlations—HR

**Figure 9:**
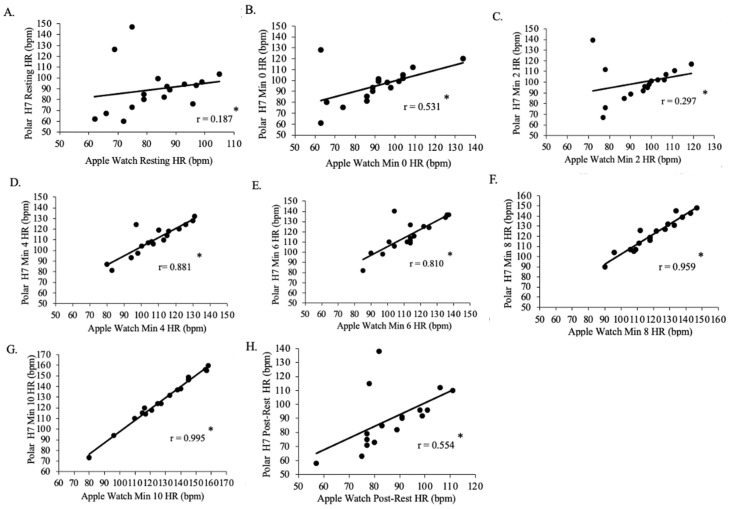
The relationship between the Apple Watch Series 9 and Polar H7 on measures of heart rate (HR) for rest (panel **A**; r = 0.187; *p* = 0.093), 0 min (panel **B**; r = 0.531; *p* = 0.006), 2 min (panel **C**; r = 0.297; *p* = 0.048), 4 min (panel **D**; r = 0.881; *p* < 0.001), 6 min (panel **E**; r = 0.810; *p* < 0.001), 8 min (panel **F**; r = 0.959; *p* < 0.001), 10 min (panel **G**; r = 0.995; *p* < 0.001), and post-rest (panel **H**; r = 0.554; *p* = 0.002) in the Group 2 participants in the study (n = 17). Data presented as values. * Statistical significance *p* < 0.05.

There was a positive weak relationship between resting Polar H7-measured HR and resting Apple Watch Series 9-measured HR for Group 2 (n = 17, *p* = 0.093, r = 0.187; Figure 9) and between min 2 Polar H7-measured HR and min 2 Apple Watch Series 9-measured HR for Group 2 (*p* = 0.048, r = 0.297). There was a positive moderate relationship between min 0 Polar H7-measured HR and min 0 Apple Watch Series 9-measured HR for Group 2 (*p* = 0.006, r = 0.531) and between min 10 Polar H7-measured HR and min 10 Apple Watch Series 9-measured HR for Group 2 (*p* = 0.002, r = 0.554). There was a positive strong relationship between min 4 Polar H7-measured HR and min 4 Apple Watch Series 9-measured HR for Group 2 (*p* < 0.001, r = 0.881) and between min 6 Polar H7-measured HR and min 6 Apple Watch Series 9-measured HR for Group 2 (*p* = < 0.001, r = 0.810). There was a positive, very strong relationship between min 8 Polar H7-measured HR and min 8 Apple Watch Series 9-measured HR for Group 2 (*p* < 0.001, r = 0.959).

#### 3.4.3. Group 3 Correlations—HR

**Figure 10:**
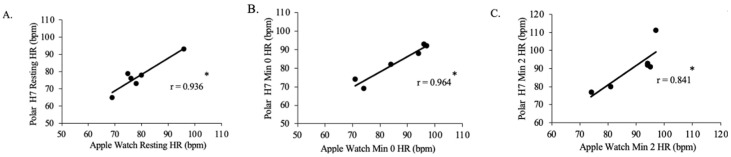

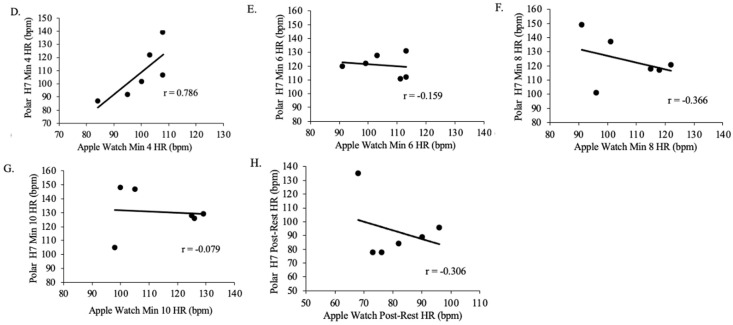
The relationship between the Apple Watch Series 9 and Polar H7 on measures of heart rate (HR) for rest (panel **A**; r = 0.936; *p* = 0.006), 0 min (panel **B**; r = 0.964; *p* = 0.002), 2 min (panel **C**; r = 0.841; *p* = 0.050), 4 min (panel **D**; r = 0.786; *p* = 0.064), 6 min (panel **E**; r = −0.159; *p* = 0.763), 8 min (panel **F**; r = −0.336; *p* = 0.658), 10 min (panel **G**; r = −0.079; *p* = 1.000), and post-rest (panel **H**; r = −0.306; *p* = 0.827) in the Group 3 participants in the study (n = 6). Data presented as values. * Statistical significance *p* < 0.05.

There was a negative negligible relationship between min 10 Polar H7-measured HR and min 10 Apple Watch Series 9-measured HR for Group 3 (n = 6, *p* = 1.000, r = −0.079; Figure 10). There was a negative weak relationship between min 6 Polar H7-measured HR and min 6 Apple Watch Series 9-measured HR for Group 3 (*p* = 0.763, r = −0.159), between min 8 Polar H7-measured HR and min 8 Apple Watch Series 9-measured HR for Group 3 (*p* = 0.658, r = −0.366), and between post-rest Polar H7-measured HR and post-rest Apple Watch Series 9-measured HR for Group 3 (*p* = 0.827, r = −0.306). There was a positive strong relationship between min 2 Polar H7-measured HR and min 2 Apple Watch Series 9-measured HR for Group 3 (*p* = 0.050, r = 0.841) and between min 4 Polar H7-measured HR and min 4 Apple Watch Series 9-measured HR for Group 3 (*p* = 0.064, r = 0.786). There was a positive, very strong relationship between resting Polar H7-measured HR and resting Apple Watch Series 9-measured HR for Group 3 (*p* = 0.006, r = 0.936) and between min 0 Polar H7-measured HR and min 0 Apple Watch Series 9-measured HR for Group 3 (*p* = 0.002, r = 0.964).

### 3.5. Linear Regressions—EE and HR

**Figure 11:**
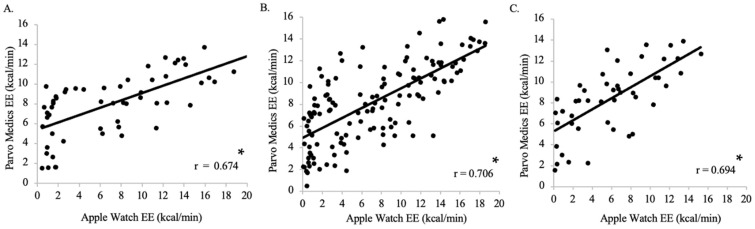
The relationship between the Apple Watch Series 9 and Parvo Medics on measures of energy expenditure (EE) for all time points for Group 1 (**A**; r = 0.674; *p* = < 0.001), Group 2 (**B**; r = 0.706; *p* = < 0.001), and Group 3 (**C**; r = 0.694; *p* < 0.001). Data presented as values. * Statistical significance *p* < 0.05.

There was a positive moderate relationship between Parvo Medics-measured EE and Apple Watch Series 9-measured EE for Group 1 (n = 7, r = 0.674), with a regression coefficient of 0.454 (*p* < 0.001), indicating that for every one kcal change, there is a 0.454 change in Apple Watch Series 9-measured EE (Figure 11A). There was a positive, very strong relationship between Parvo Medics-measured EE and Apple Watch Series 9-measured EE for Group 2 (n = 17, r = 0.706), with a regression coefficient of 0.498 (*p* < 0.001), indicating that for every one kcal change, there is a 0.498 change in Apple Watch Series 9-measured EE (Figure 11B). There was a positive moderate relationship between Parvo Medics-measured EE and Apple Watch Series 9-measured EE for Group 3 (n = 6, *p* < 0.001, r = 0.694), with a regression coefficient of 0.482 (*p* < 0.001), indicating that for every one kcal change, there is a 0.498 change in Apple Watch Series 9-measured EE (Figure 11C).

**Figure 12:**
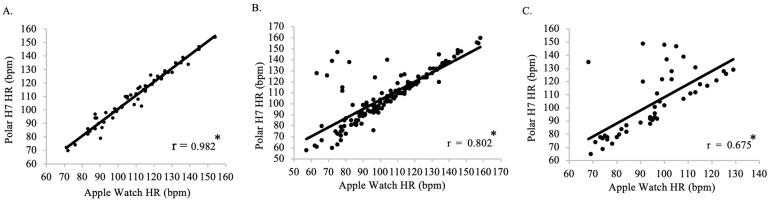
The relationship between the Apple Watch Series 9 and Polar H7 on measures of heart rate (HR) for all timepoints for Group 1 (**A**; r = 0.982; *p* < 0.001), Group 2 (**B**; r = 0.802; *p* < 0.001), and Group 3 (**C**; r = 0.675; *p* < 0.001). Data presented as values. * Statistical significance *p* < 0.05.

There was a positive, very strong relationship between Polar H7-measured HR and Apple Watch Series 9-measured HR for Group 1 (n = 7, r = 0.982), with a regression coefficient of 0.964 (*p* < 0.001), indicating that for every one bpm change, there is a 0.965 change in Apple Watch Series 9-measured HR (Figure 12A). There was a positive, very strong relationship between Polar H7-measured HR and Apple Watch Series 9-measured HR for Group 2 (n = 17, r= 0.802), with a regression coefficient of 0.644 (*p* < 0.001), indicating that for every one bpm change, there is a 0.644 change in Apple Watch Series 9-measured HR (Figure 12B). There was a positive moderate relationship between Polar H7-measured HR and Apple Watch Series 9-measured HR for Group 3 (n = 6, r = 0.675), with a regression coefficient of 0.456 (*p* < 0.001), indicating that for every one bpm change, there is a 0.456 change in Apple Watch Series 9-measured HR (Figure 12C).

## 4. Discussion

The purpose of this study was to evaluate the accuracy of the Apple Watch Series 9 for measures of EE and HR on individuals with varying skin pigmentations and whether the relation between devices was impacted by skin pigmentation. We hypothesized that the Apple Watch Series 9 is not an accurate measurement device for EE and HR and, indeed, found differences between the Apple Watch Series 9 and referent devices. Further, it was hypothesized that the optical sensor-based Apple Watch Series 9 is less accurate for darker skin pigmentations (Group 3) compared to lighter skin pigmentations (Group 1 and 2) in measures of EE and HR and revealed that relations were modified according to skin pigmentation group. Our findings reveal inconsistent results regarding the impact of self-reported skin pigmentation on the accuracy of the latest Apple Watch Series 9 in measuring EE and HR. Therefore, Apple Watch Series 9 users must be mindful that health data regarding EE and HR are rough estimates that may underestimate the actual measurements. Our findings are also meaningful for Apple, as Apple should be aware of these discrepancies. It is our hope that these findings will lead to the refinement of this technology in order for these devices to be accurate for all individuals, regardless of their skin pigmentation (or those with tattoos), to combat health disparities.

### 4.1. The Accuracy of Apple Watch Series 9 in Estimating Energy Expenditure

The findings of this study support the existing literature on the inaccuracy of Apple Watches on measures of EE and HR. A study by Duking et al. assessed the accuracy of the Apple Watch Series 4 along with other fitness tracking devices on measures of EE and HR [18]. The participants were tested similarly to this study as they exercised at various intensities. The study found that the Apple Watch was not accurate in measuring EE but was accurate in measuring HR [18]. To the best of our knowledge, no previous literature has examined the impact of skin pigmentation on measures of EE. However, research has found that EE can be indirectly measured using HR data [11].

### 4.2. The Accuracy of Apple Watch Series 9 in Estimating Heart Rate

Many studies have shown that Apple Watch-measured HR is accurate; however, these studies did not look at the impact of skin pigmentation [18,19]. Previous literature on the impact of skin pigmentation on the accuracy of Apple Watch HR measurement has been variable [20,21]. A study by Sanudo et al. found that the correlation between Apple Watch and Polar HR was very good, but there were significant differences between skin type group II and III as the intensity increased [21]. However, these differences were <2% of relative differences between devices [21]. Overall, the Apple Watch measured HR accurately compared to Polar HR when exercising at different intensities, and skin pigmentation did not seem to influence these measures [21], which was inconsistent with our study’s findings. However, a review by Colvonen et al. found that different skin pigmentations can affect light absorption, interfering with the PPG algorithm’s output, as PPG uses a green light signal to work [20]. Studies have found that green light absorption is not as accurate for measuring HR for individuals of darker skin pigmentations, which was consistent with our findings.

### 4.3. Experimental Considerations

The limitations of this study include participant population and sample size. The participants were recruited from the Skidmore College community, where white students comprise 63% of the total student body [22]. Most of the participants in this study fell into skin pigmentation Group 2 after completing the Fitzpatrick skin survey. Furthermore, the sample was small within skin pigmentation Groups 1 and 3, consisting of fewer than ten participants. Another limitation of the present study was Apple removing the oxygen saturation function from the Apple Watch Series 9. Assessing the oxygen saturation function of the Apple Watch Series 9 would have been an interesting aspect of this study, especially considering inaccuracies between skin pigmentations reported in the past regarding pulse oximeters [9].

Sources of error stem from the Polar H7 HR monitor and the Fitzpatrick scale. In some cases, the Polar H7 HR monitor provided inconsistent readings, where some participants’ HR jumped to 200 bpm after being at 80 bpm moments before. In addition, these inconsistent readings appeared to occur more in male participants, but this was not formally assessed. Therefore, such unreliable readings could have skewed the data when using the Polar H7 to compare to the Apple Watch Series 9 for HR measures; we felt it important to highlight that even referent devices may experience issues. Another source of error is the subjectiveness, or self-reported nature, of the Fitzpatrick scale. Each participant rated themselves based on the scale, leading to the possibility of participants being placed in groups that a trained dermatologist would not have put them in. This subjective grouping could have affected how we assessed the data between skin pigmentations. Future studies might directly measure skin pigmentation intensity at the measurement site, as well as those with manipulated skin pigmentation (e.g., tattoos, tanning (UV or spray), etc.).

Recommendations for future studies include having a larger, more diverse sample size, using other exercise modes, such as cycling, and looking at how different intensities of exercise impact Apple Watch Series 9 accuracy. Another recommendation is to use a 12-lead EKG as the measure criterion for HR, as this is the gold standard, and our study found that the polar H7 chest strap was inconsistent in some cases, particularly in males. In addition, it would be beneficial to look at the differences between males and females. One final area for future study could be assessing the accuracy of the Apple Watch Series 9 in lean versus obese individuals; it would be interesting to evaluate if levels of subcutaneous fat around the wrist could impact the accuracy of these devices.

## 5. Conclusions

Our study revealed inconsistencies in the Apple Watch Series 9 compared to the measure criterion for the measures of EE and HR across intensity and skin pigmentation. Overall, EE was found to be inconsistent for all skin pigmentation groups. However, for HR, Group 1′s measurements were less variable than those in Group 3, showing that PPG sensors are not as precise for darker skin pigmentations. Our findings suggest that some caution is warranted for consumers, and they should be aware of these discrepancies when considering purchasing these devices to track their health, as they may provide inaccurate data.

## Figures and Tables

**Table 1:** Descriptive characteristics of the Skidmore College participants in the study.

	Group 1 (n = 7)	Group 2 (n = 17)	Group 3 (n = 6)	All Participants (n = 30)	*p*-Value	Significant (Y or N)
Age, yrs	20.7 ± 1.4	20.9 ± 1.5	20.2 ± 1.0	20.7 ± 1.4	0.517	N
Height, cm	170.7 ± 3.7	168.1 ± 9.3	170.0 ± 6.4	169.1 ± 7.7	0.644	N
Weight, kg	68.1 ± 8.0	70.7 ± 13.8	72.0 ± 2.6	70.4 ± 11.0	0.485	N
Body Fat, %	24.1 ± 7.8	23.4 ± 7.2	22.4 ± 7.4	23.4 ± 7.1	0.915	N
Sex (M/F)	1/6	6/11	4/2	11/19	0.175	N

Values are means ± SD. Group (1) (Fitzpatrick I, II); (2) (Fitzpatrick III, IV); (3) (Fitzpatrick V, VI).

**Table 2:** Comparison between Parvo Medics and Apple Watch Series 9 on measures of EE.

	ParvoMedics (kcal/min) (N = 30)	Apple EE (kcal/min) (N = 30)	*t*-Test (*p*-Value)	Significant (Y or N)
Rest	7.1 ± 1.5	1.2 ± 0.9	<0.001	Y
0 min	2.6 ± 0.9	1.1 ± 0.9	<0.001	Y
2 min	5.3 ± 1.0	6.7 ± 2.9	<0.001	Y
4 min	8.2 ± 1.4	8.9 ± 2.7	0.136	N
6 min	9.7 ± 1.7	10.0 ± 3.5	0.554	N
8 min	11.1 ± 1.9	11.8 ± 3.8	0.293	N
10 min	12.5 ± 2.2	13.3 ± 4.1	0.258	N
Post	9.1 ± 1.6	2.4 ± 1.2	0.001	Y

Values are as means ± SD.

**Table 3:** Comparison between Polar H7 and Apple Watch Series 9 on measures of HR.

	Polar H7 (bpm) (N = 30)	Apple HR (bpm) (N = 30)	*t*-Test (*p*-Value)	Significant (Y or N)
Rest	86.9 ± 18.3	83.7 ± 12.0	0.736	N
0 min	94.3 ± 15.9	91.7 ± 16.4	0.523	N
2 min	98.7 ± 14.7	96.1 ± 12.8	0.212	N
4 min	110.7 ± 14.0	106.9 ± 13.0	0.007	Y
6 min	118.1 ± 13.1	112.7 ± 13.3	0.020	Y
8 min	124.2 ± 14.9	119.8 ± 15.7	0.054	N
10 min	130.2 ± 19.4	127.0 ± 19.3	0.271	N
Post	89.8 ± 18.6	85.6 ± 12.2	0.819	N

Values are as means ± SD.

**Table 4:** Comparison between Parvo Medics and Apple Watch Series 9 on measures of EE for each Group.

	ParvoMedics (kcal/min)	Apple EE (kcal/min)	*t*-Test (*p*-Value)	Significant (Y or N)
Group 1 (n = 7)	8.1 ± 3.2	7.2 ± 5.4	0.168	N
Group 2 (n = 17)	8.2 ± 3.2	7.2 ± 5.0	0.011	Y
Group 3 (n = 6)	8.4 ± 3.3	5.9 ± 3.8	<0.001	Y

Values are means ± SD.

**Table 5:** Comparison between Polar H7 and Apple Watch Series 9 on measures of HR for each Group.

	Polar H7 (bpm)	Apple HR (bpm)	*t*-Test (*p*-Value)	Significant (Y or N)
Group 1 (n = 7)	111.9 ± 18.3	111.5 ± 17.7	0.088	N
Group 2 (n = 17)	106.4 ± 14.7	103.3 ± 16.5	0.739	N
Group 3 (n = 6)	103.4 ± 20.1	95.1 ± 13.1	0.014	Y

Values presented as means ± SD.

## Data Availability

Data available upon reasonable request of the corresponding author.
